# Automation and Scale Up of Somatic Embryogenesis for Commercial Plant Production, With Emphasis on Conifers

**DOI:** 10.3389/fpls.2019.00109

**Published:** 2019-02-18

**Authors:** Ulrika Egertsdotter, Iftikhar Ahmad, David Clapham

**Affiliations:** ^1^Department of Forest Genetics and Plant Physiology, Umeå Plant Science Centre, Swedish University of Agricultural Sciences, Umeå, Sweden; ^2^G.W. Woodruff School of Mechanical Engineering, Georgia Institute of Technology, Atlanta, GA, United States; ^3^Department of Plant Biology, Uppsala BioCenter, Swedish University of Agricultural Sciences, Uppsala, Sweden

**Keywords:** somatic embryogenesis, conifer, automation, scale up, bioreactors

## Abstract

For large scale production of clonal plants, somatic embryogenesis (SE) has many advantages over other clonal propagation methods such as the rooting of cuttings. In particular, the SE process is more suited to scale up and automation, thereby reducing labor costs and increasing the reliability of the production process. Furthermore, the plants resulting from SE closely resemble those from seeds, as somatic embryos, like zygotic (seed) embryos, develop with good connection between root and shoot, and without the plagiotropism often associated with propagation by cuttings. For practical purposes in breeding programs and for deployment of elite clones, it is valuable that a virtually unlimited number of SE plants can be generated from one original seed embryo; and SE cultures (clones) can be cryostored for at least 20 years, allowing long-term testing of clones. To date, there has however been limited use of SE for large-scale plant production mainly because without automation it is labor-intensive. Development of automation is particularly attractive in countries with high labor costs, where conifer forestry is often of great economic importance. Various approaches for automating SE processes are under investigation and the progress is reviewed here, with emphasis on conifers. These approaches include simplification of culture routines with preference for liquid rather than solid cultures, use of robotics and automation for the harvest of selected individual mature embryos, followed by automated handling of germination and subsequent planting. Different approaches to handle the processes of somatic embryogenesis in conifers are outlined below, followed by an update on efforts to automate the different steps, which are nearing an operational stage.

## Introduction to Somatic Embryogenesis for Large-Scale Plant Production

Somatic embryogenesis is a desirable method for clonal propagation of plants as it offers many biological and practical advantages. Biologically, the somatic embryo progresses through a developmental process much like its zygotic counterparts (Pullman et al., 2003; Cairney and Pullman, 2007). Development of root and shoot meristems results in good connection between root and shoot in the resulting plant, avoiding the issues with plagiotropic growth, and adventitious root formation that are concerns with propagation by cuttings. Furthermore, the opportunity to keep embryogenic cell lines for prolonged periods in cryostorage allows for field-testing before selection of production cell lines (Park et al., 2014). For commercial plant production, especially the earliest *in vitro* phase of embryo multiplication offers opportunities to scale up for bulk production of desirable genotypes by using liquid culture medium.

Various private companies have estimated the economics of *in vitro* plant production. One of the few public estimates completed already in the mid-1990s showed that about 50% of the production costs for *in vitro* plants stem from labor (Cervelli and Senaratna, 1995). As labor costs of the various steps of the process are high, cost components could change dramatically by for example automating SE multiplication in bioreactors (Cervelli and Senaratna, 1995; Heyerdahl et al., 1995). Still, more than 20 years later, reviews point to the high cost of labor as prohibitive for large scale plant production in the absence of automation (Lelu-Walter et al., 2013). This applies particularly to conifers, the most important forest species in the northern hemisphere.

**Figure 1:**
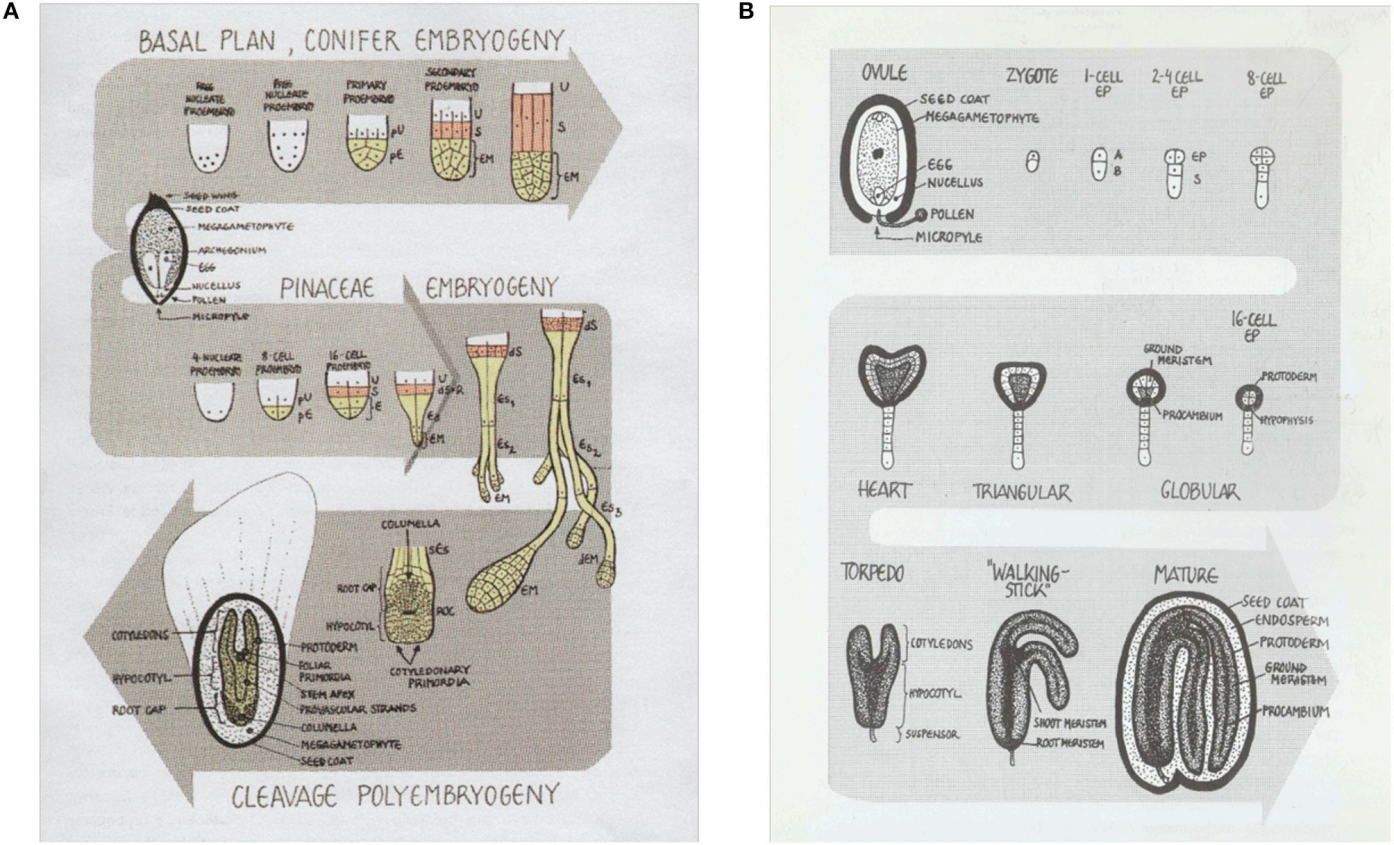
Zygotic embryo development in gymnosperms and angiosperms differs in some major aspects. **(A)** Early stages of conifer embryo development. The conifer zygote results from a single fertilization event. At the beginning of embryogenesis, there are free nuclear stages followed by a pro-embryo stage. Main characteristics of conifer embryo development are the polyembryogenic features of some conifer species where the zygotic embryos to different degrees proceed through a process of embryo-cleavage that results in multiple embryos that are eventually eliminated by programmed cell death. **(B)** In angiosperm embryo development the sporophytic generation is initiated by a double fertilization event resulting in one embryo. **(A)** pU, primary upper tier; pE, primary embryonal tier; U, upper tier; S, suspensor tier; EM, embryo mass; dS, R: dysfunctional suspensor tier; Es, embryonal suspensor tier; dEM, degenerating embryo mass; ROC, root organization center; sEs, secondary embryonal suspensor cells. **(B)** A, apical cell; B, basal cell; EP, embryo proper; S, suspensor. Figure adapted from Egertsdotter (1996).

**Figure 2:**
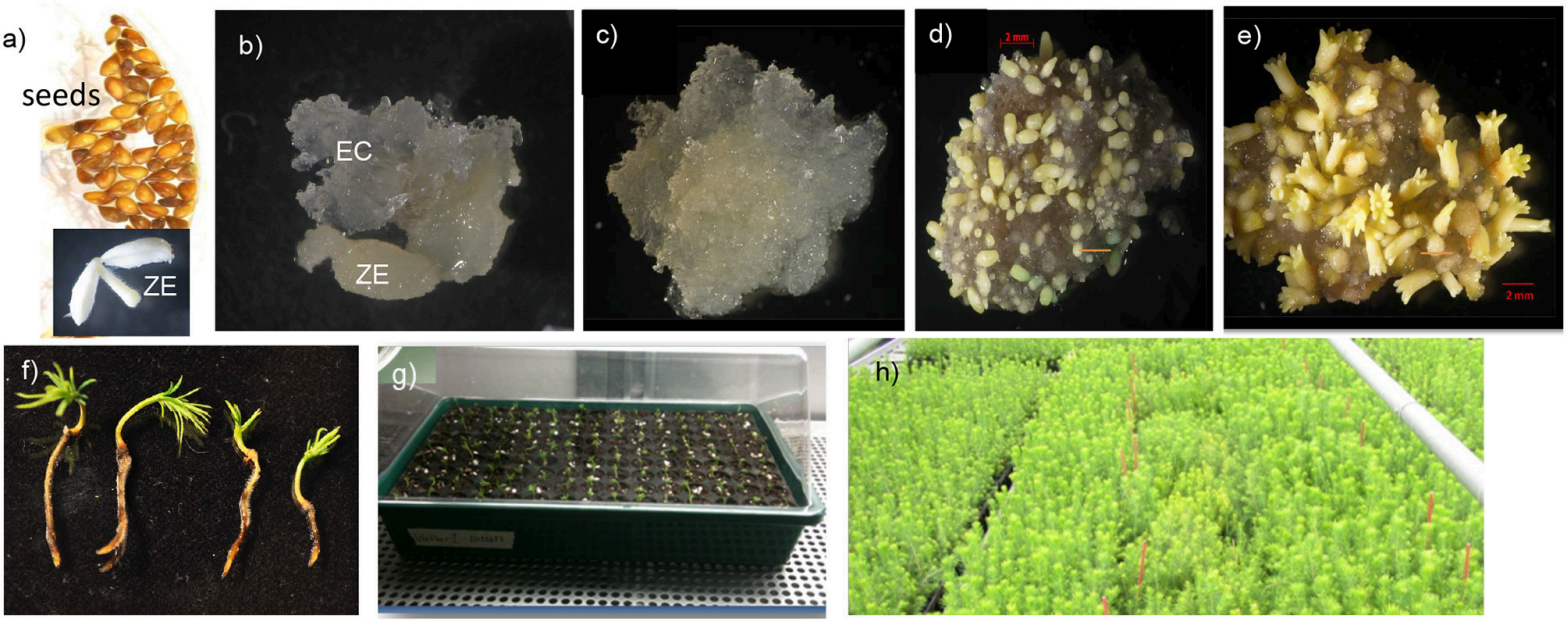
Overview of the SE process in Norway spruce (similar in other conifers). **(a)** Seeds from selected crossings are surface sterilized and used to extract zygotic embryos (ZE). Zygotic embryos are put in contact with initiation medium. **(b)** Embryogenic “callus” (EC) composed of early-stage somatic embryos (proembryogenic masses) protrudes from the zygotic embryo and continues to multiply to form a callus-like culture that can be isolated onto new medium **(c)**. After transfer of the multiplying culture to maturation medium, multiplication stops and maturation starts **(d)**. Fully mature embryos **(e)** can be isolated and transferred to desiccation before stimulated to develop into germinants with sufficient root and shoot development for transfer to *ex vitro* conditions **(f)**. SE plants develop after a period of acclimatization **(g)** and can then grow under regular nursery conditions **(h)**. Figure adjusted after Egertsdotter (2018). Scale bars represent 2 mm.

Somatic embryos are induced directly from different parts of the plant depending on species or indirectly from undifferentiated callus tissue (Halperin, 1995; Yeung, 1995; Fehér et al., 2003). Differences in the pathway of zygotic embryogenesis between angiosperms and conifers, as outlined in Figure 1, are reflected in the corresponding pathways of somatic embryogenesis. The basic laboratory processes supporting somatic embryogenesis are, however, largely similar across species in terms of the steps of the *in vitro* processes required to produce a plant: after the first step of initiating the somatic embryos from the explant, follow the cultural handling steps of multiplication, embryo development, germination, and plant formation after transfer to *ex vitro* for plant establishment in compost (Figure 2). In the present review, the somatic embryogenesis processes from early stage somatic embryos, to planting *ex vitro* of the germinated embryos, will be discussed. In an earlier review on the same topic (Ibaraki and Kurata, 2001) that focused on angiosperm SE, the main processes outlined were (1) induction, (2) maintenance of embryogenic cultures and (3) development of embryos. In this present review, the focus will be on automation and scale up of the various steps required to produce a plant from a somatic embryo in conifer species, emphasizing progress since 2001 that has been accomplished particularly with the economically important Norway spruce (*Picea abies* L. Karst).

To date, the majority of somatic embryo cultures in conifer species have been induced from seed embryos. To characterize and score the resulting trees from the SE cell lines initiated from a seed, most commercial entities are deploying SE plants into field testing whilst keeping the corresponding SE cultures in cryogenic storage. Another approach to clonal mass propagation, at the same time integrating somatic embryogenesis into the traditional breeding program, is via “family forestry” (Rosvall et al., 1998; Lindgren, 2008): the mass propagation of untested individuals or clones from tested families, i.e., families that have already been tested in the course of the breeding program. Planting with a mixture of 25 clones from six parents of tested families at each stand removes the dangers of excessive genetic uniformity associated with single clones, while exploiting the full genetic gains at the family level and avoiding the considerable expense and delay associated with effective clonal testing (Lindgren, 2008). In Scandinavia trees must be at least 10 years old before selection for growth characters is meaningful, owing to poor juvenile/mature correlations; but this situation may change with the development of mass genomic selection (e.g., Iwata et al., 2011; Beaulieu et al., 2014)

As SE plant production from at least Norway spruce may become a reality in the near future owing to the recent technological developments, the possible impact from integration of SE plants as parts of clonal forestry operation into Swedish forestry was recently examined from different perspectives. The benefits in terms of genetic gains and the potential risks of clonal forestry were reviewed by Wu (2018), and effects of clonal forestry on genetic diversity in wild and domesticated stands of forest trees by Ingvarsson and Dahlberg (2018). Furthermore, the potential inherent risks from the SE process were examined by Egertsdotter (2018). Taken together, it can be concluded that with sufficient numbers of clones in production, that are replaced at certain intervals, SE plants can soon become a cost-effective, accepted and viable contribution to Swedish forestry operations provided that the required automation becomes reliably available.

## Overview of Somatic Embryogenesis in *in vitro* Developmental Processes

### Initiation

The starting operation of initiating an SE-culture is typically associated with detailed dissection of certain cell-layers or structures of the responsive tissue in an initial explant. There are no known efforts directed toward automation of this process. In conifers, where the common source of initial explant is the zygotic embryo, extraction of the female megagametophyte (FMG), and even extraction of the ZE from within the FMG, are required for success. With the diversity of seed sizes and shapes, and the sensitivity of the target tissue (the embryo), this process has not been an attractive target for automation. Also, for many angiosperms where the initial (vegetative) explant is accessible and plentiful, other parts of the SE process are more relevant for automation.

### Multiplication

After establishment from the initial explant, the conifer SE culture is maintained or proliferated by repeated subculture into fresh media promoting multiplication. Auxins and cytokinins are common plant growth regulators (PGRs) that enable proliferation of early stage embryos (e.g., von Arnold and Clapham, 2008) and the nature of the nitrogen source is important (e.g., Carlsson et al., 2017). Liquid suspension cultures of conifers can multiply at higher rates and with less aggregation than those on solid medium (Von Arnold et al., 2002) and are consequently preferred for the multiplication phase. Agitation of the liquid medium helps to break up the PEM tissue into smaller aggregates of PEMs that can multiply without hindrance from connected tissue (Egertsdotter and von Arnold, 1998). Multiplication in liquid medium also enables scale up in bioreactors and automation of the process. However, for the subsequent maturation, full immersion in liquid medium does not work effectively, as discussed below. The time in the multiplication phase is determined by the rate of multiplication of the particular species and cell line, and the target number of plants to be produced. In Norway spruce, the composition of the multiplying culture in terms of proportion of early stage somatic embryos, or stages of the proembryogenic masses (PEMs) present that can respond to the maturation treatment and continue to the next developmental stage, has been carefully investigated at the cellular level (Filonova et al., 2000a,b). The proportion of PEMs responsive to maturation treatment greatly influences the yield from conifer SE cultures. In angiosperms, the same diversity of developmental stages of early stage multiplying embryos can be observed. Owing to the morphology of the somatic embryos, fractionation of sizes by sieving the cultures has been shown to support synchronization in embryo cultures in angiosperms, e.g., in *Daucus carota* L. (Fujimura and Komamine, 1979), *Citrus sinensis* (L.) Osbeck (Souza et al., 2011), and *Fraxinus angustifolia* Vahl (Tonon et al., 2001). As the early stage embryos of a conifer PEM culture are composed of tightly associated clusters of embryos forming a web of embryonic tissue, fractionation and sieving of different stages of conifer somatic embryos are not effective.

### Maturation

In conifers, maturation of somatic embryos is triggered by a change in the culture medium composition where PGRs and carbohydrates are key components (e.g., von Arnold and Clapham, 2008). Typically, maturation starts on a medium without auxin and cytokinin but supplied with abscisic acid, a key regulator (Tikkinen et al., 2018a). Maturation usually takes at least 4 to 6 weeks depending on species and cell line. Embryos mature asynchronously, partly reflecting their previous developmental history. Through dispersion and by addition of suitable carbohydrates, synchronization of mature embryos can be increased (Egertsdotter and Clapham, 2011). To date, most conifer SE cultures are matured placed directly in contact with solidified medium or on a filter paper on the solid medium surface if plated out from a suspension culture. Efforts to produce mature embryos continuously immersed in liquid medium in bioreactors or suspension flasks have not encouraged large-scale production except for particular cell lines of white spruce, *Picea glauca* (Moench) Voss (Hakman and von Arnold, 1988) and Norway spruce (Gorbatenko and Hakman, 2001). Maturation on solid medium in plates or other vessels allows formation of mature embryos from the PEMs of many cell lines, and is still the most commonly used method for embryo maturation. However, embryo maturation can also be obtained in temporary immersion bioreactors where the embryos rest on a solid support and are fed intermittently with maturation medium (Businge et al., 2017; Mamun et al., 2018). When maturation is complete, embryos are often subjected to a desiccation treatment in preparation for storage and germination. Automated processes for maturation based on a solid support in temporary immersion bioreactors have been described for various conifer species and are discussed below.

### Germination and Plant Formation

Mature embryos are typically desiccated under high relative humidity. Desiccation for extended times at lower temperatures was recently shown to give increased rates of germination (Tikkinen et al., 2018b). The conditions for successful desiccation allowing for long-term storage of mature embryos have been carefully investigated (Attree and Fowke, 1993 and references therein). The storage can help to accumulate embryos from recalcitrant cell lines and to adapt the germination time to other nursery operations. But if controlled environments are available, established plants, inwintered as appropriate, can be stored according to conventional nursery practices as widely applied for Norway spruce and Scots pine (*Pinus sylvestris*) in Sweden (Ersson, 2016).

Germination is started by placing the mature (desiccated) embryo in contact with germination medium. Most commonly, this is gelled germination medium in a petri plate, but efforts have been made to supply liquid germination medium to mature embryos placed on a porous material for the purpose of scaling up the process (discussed below). Darkness or dim red light are often recommended for the first 2 to 3 weeks of germination (Kvaalen and Appelgren, 1999) which often proceeds for 6 weeks or longer (Dobrowolska et al., 2017); but recently, appropriate cold pre-treatments were shown to improve germination rates after only 1 week of *in vitro* germination (Tikkinen et al., 2018a,b). When sufficient root and shoot have developed, the germinants are planted in compost and gradually acclimatized to suitable *ex vitro* conditions based on experience with seedlings of various provenances grown in controlled environments; for example, continuous light at 20°C for *Picea abies*, long days and short nights (nights at reduced temperature) for boreal *Pinus* species (Ekberg et al., 1979; Högberg et al., 2001).

## Automation of Different Steps of the SE Process

### Automation and Bioreactors for Scale Up of Embryo Production

The multiplication step, possible in liquid medium with most species, offers the choice of scale up in bioreactors and thereby automation of the process. The tendency in the laboratory has been for all steps of the process to take place in petri plates. Automated production of the petri plates with solid medium can be applied to reduce labor, but transfer of the SE cultures between plates is still manual even in larger scale operations (e.g., KF Bioplants, Pvt, Ltd.,Pune, India). For multiplication, there are options for scale up by culture in liquid medium in suspension flasks or different types of bioreactors (Mamun et al., 2015). Liquid medium has many advantages in terms of providing the propagules better and more even access to medium components as well as facilitating automation. One major down side is that liquid-based cultures are more prone to contamination than those on solid medium, which is especially daunting for large-scale bioreactor cultures.

The conifer SE processes differ in some ways from the angiosperm SE processes. Importantly for scale up processes in bioreactors, conifers appear to prefer a solid substrate to support directional growth of the most developmentally advanced PEMs (Sun et al., 2010). A polarized early stage PEM structure which represents the more developed early stages of somatic embryos is most likely to respond to the maturation treatment (Sun et al., 2011). Therefore, temporary immersion bioreactors providing solid support for culture growth by the mesh constituting the bottom of the bioreactor insert promotes directional growth of PEMs and formation of polarized early stage embryos and can thus have advantages over other types of bioreactors based on complete immersion and agitation of the culture.

**Figure 3:**
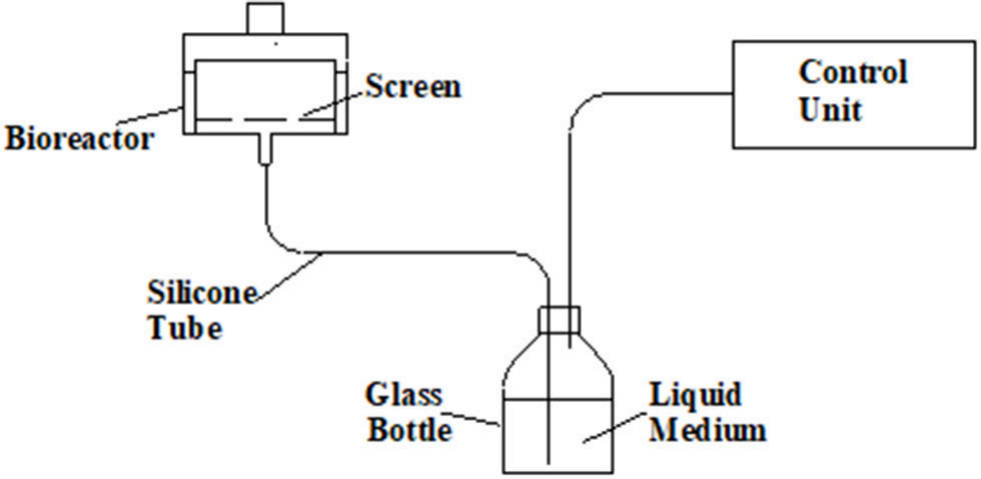
Temporary immersion bioreactor model with the liquid medium separated from the culture container. Feeding is controlled by a digital timer through a solenoid valve.

Temporary immersion bioreactors can provide the required conditions for both multiplication and maturation of conifer somatic embryo development. Such bioreactors can be used for the shoot multiplication of hardwood trees (Businge et al., 2017) and probably for the germination of conifer somatic embryos. The bioreactor can have separate containers for plant material and culture medium, allowing for easy and safe exchange of culture medium without disturbing the culture (Figure 3); or, the culture medium is held within the same container as the culture, separated by a screen, e.g., RITA bioreactor types (VITROPIC). For SE cultures of conifers, multiplication and maturation can occur sequentially in the same bioreactor without disturbing the culture. Bioreactors are started with PEMs cultures grown as suspension cultures, or solid cultures that are dispersed at the start of the bioreactor culture (see details below).

Multiplication of conifer PEMs results in clumps of connected tissue where the interior parts become dormant over time. To optimize multiplication rates and development of PEMs, it is beneficial to create a thin layer of PEMs where a larger number of early stage embryos are exposed to culture medium. Such growth conditions can be created by dispersing the clumps of PEMs into a suspension and distributing the suspension onto a supportive medium surface (Aidun, 2015; Aidun and Egertsdotter, 2015). Increasing the proportion of active PEMs also increases the number of mature embryos that can be formed and thereby the conversion rate of mature embryos from PEMs. This procedure is particularly useful for culture in bioreactors based on liquid culture medium (Mamun et al., 2015). However, to date the successful use of temporary immersion bioreactors has been reported only for *Abies nordmanniana* (Businge et al., 2017) and Norway spruce (Aidun and Egertsdotter, 2012; Mamun et al., 2018) using the model with separate container for medium that reduces humidity in the culture area (Figure 3). The immersion time was adjusted to allow the liquid medium to immerse the cultures, then to immediately retract without soaking. This typically results in immersions of 15–30 s duration. For multiplication and maturation, standard settings for immersions are 2–4 times every 24 h, depending on cell line.

Efforts toward automation and scale up of the bioreactor process for multiplication of conifer SE cultures have been attempted with bioreactors based on the same principal as the single-use wave-mixed bag-system in use for mammalian cell culture (Swanda, 2015a; single-use bioreactors reviewed in Eibl et al. (2010); use for plant cell culture in general reviewed in Eibl et al. (2018). In brief, such bioreactors are based on full immersion of the culture in liquid, enclosed in a plastic bag. The bag is placed on a moving platform to create movement of the liquid for aeration. Similarly to the mammalian use bioreactors, the bioreactor system intended primarily for conifers has different ports, which could be used for attaching different types of sensors (e.g., for temperature, salts, pH), or transporting culture material in or out of the bioreactor with the action of a pump. The system can also be equipped with an automated gas control (Swanda, 2015b). To date there are however no publications showing successful use of such bioreactor system for SE cultures in conifers. On the contrary, for commercial scale up of SE plant production in *Coffea canephora*, cultures are multiplied in a 10 L disposable bioreactor consisting of a bag containing a rigid plastic box. Further developments are plastic-based disposable bioreactors for 10 to 100 L working volumes of coffee, enabled by successful introduction of a bag system for production of torpedo-stage embryos (Ducos et al., 2009) In this system, full liquid-immersion plastic bag bioreactors are utilized for SE-multiplication to torpedo stage embryos, followed by production of cotyledonary embryos in temporary immersion bioreactors. Cotyledonary stage embryos are then transferred to *ex vitro* conditions and manually planted. For *Coffea arabica*, Etienne et al. (2013) designed a polycarbonate bioreactor (MATIS) similar to a RITA but of 5L volume, with a larger area allowing the spreading out of the biomass to obtain greater light transmittance. This raised the embryo-to-plantlet conversion from 55 to 91%. A major difference between the conifer and angiosperm SE process so far preventing the same processes to be successful for conifer SE plant production may be the typically required step of desiccation after production of mature (cotyledonary stage) conifer somatic embryos, and the apparent benefit on germination from cleaning off PEMs and medium components like PEG from the mature embryos before germination.

Efforts with other angiosperm species have been mostly in temporary immersion bioreactors, e.g., RITA bioreactor types, but some other types have also been tested (examples in Table 1). Additional species and details on different bioreactor designs have been described earlier in a book focused on bioreactor utilization (Hvoslef-Eide and Preil, 2005). In a broadly aimed effort to scale up liquid SE suspension cultures, a custom-made bioreactor holding 2 L liquid medium and equipped with stirrers and oxygenation was tested. Increased rates of multiplication rates were observed for embryogenic cultures of carrot (*Daucus carota*), Norway spruce (*Picea abies*), birch (*Betula pendula*), cyclamen (*Cyclamen persicum*), and shoot cultures of Christmas begonia (*Begonia x cheimantha*) in custom-made bioreactors (Hvoslef-Eide et al., 2005).

**Table 1:** Representative published articles on the utilization of bioreactors for SE-based plant production in angiosperms.

**Species**	**Type of bioreactor**	**Comments on key content**	**References**
*Allium cepa* (onion)	3 L balloon type air lift, with continuous or temporary immersion	Nearly twice as fast proliferation with continuous as compared with temporary immersion	Wu et al., 2015
*Bactris gasipaes* (peach palm)	RITA and twin flask system	Higher quality PEMs giving four times as many plants obtained from RITA than from twin flasks	Heringer et al., 2014
	Temporary immersion system (twin flasks)	Induction of secondary SE was higher (84.6%) in bioreactor than on solid medium (70.2%)	Steinmacher et al., 2011
*Camellia sinensis* (tea)	Custom temporary immersion bioreactor	Four-fold higher yield of gobular embryos with TIS than obtained on semi-solid media	Akula et al., 2000
*Carica papaya* (papaya)	RITA	Up to 95% of SEs showed complete germination in RITA depending on inoculum density	Posada-Pérez et al., 2017
*Castanea dentata* (American Chestnut), *(C. dentata x Castanea mollissima)* (American-Chinese chestnut back crosses)	Airlift bioreactors (ALBs)	Higher yields of small cell clumps suitable for transformation in ALBs than in suspension flasks	Kong et al., 2014
*Citrus deliciosa*	Custom-made temporary immersion bioreactor	TIB stimulated formation of cotyledonary stage embryos, but germination still poor in all culture conditons	Cabasson et al., 1997
*Coffea arabica* L. (arabica coffee)	RITA and MATIS	Comparison of zygotic and somatic embryo germination and plant formation provides information to improve SE germination in larger bioreactors.	Etienne et al., 2013
.	RITA	Effects on yields and vitrification from immersion cycles. Six immersions/day produced more embryos (3,081) than two immersions/day (2,094)	Albarrán et al., 2005
	RITA	Demonstration of benefits from germination in RITAs for direct sowing in soil. 200 mg of embryogenic callus produced 8,000 embryos in one bioreactor. The germination frequency was 66%	Etienne-Barry et al., 1999
	RITA	800 somatic embryo per bioreactors were produced. Germination rate was 86%. A positive relation of cotyledon size of embryos germinated in RITA for plant establishment	Barry-Etienne et al., 2002
	RITA	Highest number of somatic embryos (25) obtained in bioreactor. The germination rate was 100%. SE-multiplication in suspension cultures and plant regeneration in RITA for the purpose of genetic transformation	Gatica-Arias et al., 2008
*Coffea canephora* (robusta coffee)	Temporary immersion bioreactors (TIBs)	Pilot scale process for batch-production of torpedo stage embryos in suspension flasks, germination in TIBs and transfer to *ex vitro* after storage	Ducos et al., 2007
	10 L “Box- in- bag” bioreactors, TIBs	Batch-production of torpedo stage embryos in “Box-in-bag bioreactor,” germination in TIBs and transfer to *ex vitro* after storage	Ducos et al., 2009
*Elaeis guineensis* (oil palm)	Custom-made temporary immersion system modified from Nalgene filter units	Growth rate of embryogenic callus was 0.38 g/week	Sumaryono et al., 2008
	RITA	Fresh weight of callus increased 7-fold in RITA bioreactors	Marbun et al., 2015
	RITA and twin flask system TIS	100% increase in fresh biomass in twin flask compared with RITA	Gomes et al., 2016
*Eleutherococcus senticosus* Maxim (Siberian ginseng)	Bubble column bioreactor	Higher numbers of somatic embryos and better growth developed in bioreactors than in suspension cultures	Yang et al., 2012
*Eurycoma longifolia*	RITA	Optimization of immersion intervals and demonstration of plant regeneration after RITA embryo production	Mohd et al., 2017
*Hevea brasiliensis* (rubber)	Custom-made temporary immersion bioreactor	Improved yields in all steps of the SE process from embryogeneic callus to germination	Etienne et al., 1997
*Kalopanax septemlobus*	Temporary immersion 2 L bioreactors with net, (TIN) or continuous immersion with net (CIN)	85% of embryos produced plantlets with TIN, vs. 29% with CIN	Kim et al., 2011
*Leucojum aestivum*	RITA	46% increased efffects from culture system on secondary metabolite production in somatic embryo cultures	Ptak et al., 2013
*Musa acuminata* (banana)	5 L balloon type bubble column bioreactor or shake flasks	Highest frequency (87%) normal plants obtained among regenerants after callus proliferation in a pH-controlled bioreactor	Chin et al., 2014
*Musa ssp. (banana and plantain)*	Temporary immersion system modified from Nalgene filter units, or semi-solid medium	Number of embryos muliplied 9-fold after 2 months in bioreactor, 3-fold on semi-solid medium. Subsequent germination on semisolid medium was 60–70% in both cases	Escalant et al., 1994
*Panax notoginseng*	3 L air lift bioreactor	Early globular embryos (10 g) developed into cotyledonary embryos (79.7 g) in 4 weeks in bioreactor. These converted to plantlets if treated with GA.	You et al., 2012
*Phoenix dactylifera* L. (date palm)	TIS	A mixed system including culture on semi-solid medium, reduced costs (vs. traditional methods) and the time for plantlet production reduced to < 3 years	Almusawi et al., 2017
	Custom temporary immersion system bioreactor	Formation of 172 somatic embryos per bioreactor from 500 mg embryogenic callus	Al mayahi, 2015
	RITA	Suspension cultures gave more embryos than RITA	Ibraheem et al., 2013
*Quercus robur* (common oak)	RITA	Improved proliferation and production of cotyledonary stage embryos in temporary immersion bioreactors compared to semi-solid medium; 20% increase	Mallón et al., 2012
*Quercus suber* (cork oak)	RITA	7.5 fold increase in fresh weight in RITA compared with on semisolid medium. Immersion frequency had strong influence; best I min every 4 or 6 h	Perez et al., 2013
*Saccharum officinarum* (sugar cane)	RITA	Increased yield of *in vitro* produced plants in RITA compared to semi-solid culture	Snyman et al., 2011
*Theobroma cacao* (cocoa)	Twin flask TIS bioreactors	Significantly higher numbers of embryos in TIS than on solid medium. Improved conversion rate to torpedo-shape	Niemenak et al., 2008
*Vitis amurensis*	3 L balloon-type air lift bioreactor	SE biomass growth was higher in bioreactor (328.45 g/L) than on solid medium (69.60 g/L)	Sun et al., 2016
*Vitis vinifera* L.	Air-lift bioreactor	Pro-embryogenic masses doubled in fresh weight in bioreactors compared to suspension flasks without any losses in viability and regenerative capacity	Tapia et al., 2009

Although there are many reports of positive response to culture in temporary immersion bioreactors, other types of bioreactors have sometimes been shown to pose some stress to the somatic embryo development. In barley the embryogenic potential of cultures was significantly reduced in 2 L suspension culture bioreactors with agitation speed of 60 rpm as compared with the conventional small vessels (Stirn et al., 1994). It was observed that *Ipomoea batatas* embryos were ruptured and damaged when cultured in airlift bioreactors, resulting in limited production of embryos in these bioreactors. The shear stress of flushing fresh air inside airlift bioreactor was believed to cause the embryogenic damage during somatic embryo production and development (Bienick et al., 1995).

Overall, as concluded in an earlier review (Ascough et al., 2004), liquid SE cultures in bioreactors can show better growth and proliferation of different stages of the SE culture process, and better root and shoot formation, than those on solidified media. The reason may be better availability of nutrients and water, but some problems associated with liquid cultures such as hyperhydricity and injury to somatic embryos should be addressed by improved culture conditions.

### Singularization and Harvest of Mature Somatic Embryos

**Figure 4:**
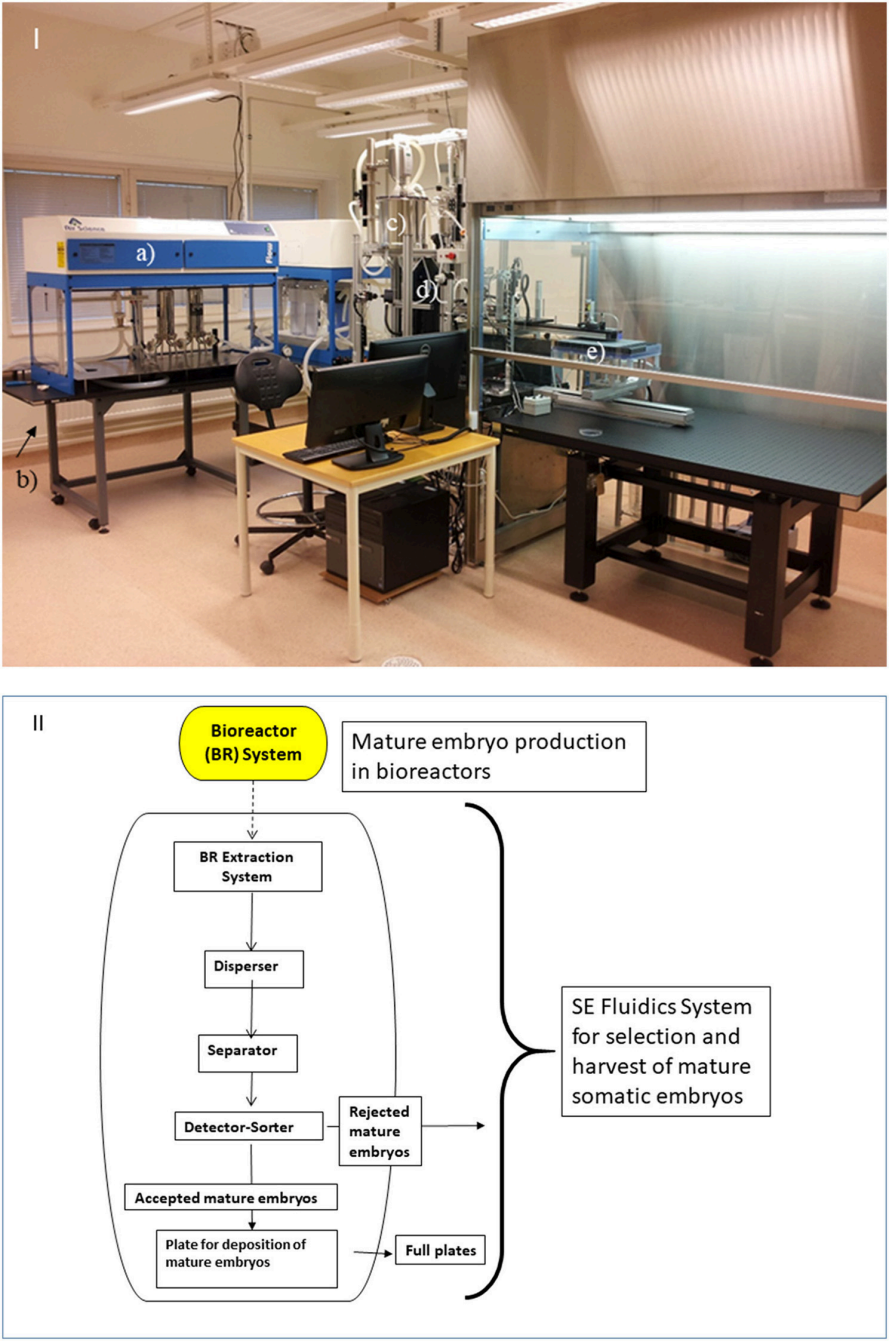
An SE Fluidics System in use for research at Umeå Plant Science Center, Swedish University of Agricultural Sciences in Umeå, Sweden. I. Overview of the SE Fluidics System and the main functional parts: The core parts of the system are: **(a)** a laminar flow hood for extraction of tissue from the bioreactors (or other culture vessels); **(b)** disperser-system that disperses the culture of PEMs and embryos; **(c)** a separator tank that separates out mature embryos for **(d)** image analysis. **(e)** Mature embryos are deposited into the container or substrate of choice. II. Flow chart showing the various steps in the SE fluidics system from proliferation and maturation in bioreactor to transfer (extraction) of the mature embryos and unresponsive PEM masses from the bioreactors into the SE Fluidics system; dispersion of the tissue from the bioreactors into mature embryos and clusters of PEMs; separation of mature embryos from the PEMs, with only mature embryos proceeding in the SE Fluidics system after the separator tank; optional image analysis of mature embryos and sorting based on pre-set selection criteria, and deposition of selected mature embryos. The system can also be run without image analysis where all mature embryos are harvested, or with image analysis where all embryos are imaged but without selection based on specific morphologies. For details of the constructions, see Aidun and Egertsdotter (2018).

When many early stage somatic embryos of the multiplying culture will not respond to the maturation treatment but remain as PEMs, it is necessary to harvest the mature embryos by separation from the PEMs. Too many decaying PEMs will affect the down-stream developmental processes of the mature embryo during germination. The harvest of individual mature embryos from the maturation-culture is arguably the most time-consuming step in the process of producing SE plants (in conifers). Automation of such a process of isolating mature embryos from the residual tissue of PEMs has also been attempted in various approaches for the purpose of scale up. One process is described that utilizes a liquid medium for spreading out isolated embryos across a porous substrate. The substrate is designed to be manipulated to accommodate the mature somatic embryos through desiccation and germination by the aid of a robotic arm moving pieces of porous material holding mature embryos (Swanda and Givens, 2017). Another more recent system, the SE Fluidics System (Aidun, 2018; Aidun and Egertsdotter, 2018) handles the SE cultures of PEMs and mature embryos in liquid through dispersion of the mature embryos and PEM tissue (Aidun, 2015; Aidun and Egertsdotter, 2015), separation of mature embryos, and an image analysis that compares individual harvested embryos to the pre-set selection criteria (Figure 4). A pilot-scale factory at SweTree Technologies in Uppsala, Sweden, based around an SE Fluidics System was run between 2014 and 2016 to demonstrate a pilot production capacity of 1 million plants/year. Current joint efforts of Swedish forestry companies and SweTree Technologies aim toward larger-scale production facilities based on a similar system with an annual commercial production of 10 million SE plants.

Robotics-based technology development was initiated for isolation, selection, and handling of mature somatic embryos for quality assessment, sorting and orientation of mature embryos of *Abies nordmanniana* and *Picea sitchensis* (Find and Krogstrup, 2008). No further development of this technology has however been reported. Robotics combined with image analyses has also been utilized for selection and handling mature embryos and germinants as part of the concept for developing an artificial seed. A system for automatically harvesting and screening mature embryos for the purpose of selecting the best embryos for insertion into artificial seeds was developed (Timmis et al., 2010). The system uses vibrational sieving, moving embryos by liquid jets and handling of embryos by robotics to sort and separate embryos according to size and shape, supported by image analysis to identify embryos most likely to germinate.

In another effort to reduce labor and build a foundation for automation and scale up by automating the step of embryo-harvest, a set up was designed for washing cultures of mature embryos free from PEMs and PEG and transfer of isolated embryos to a substrate for germination preparation (Nehra et al., 2012). Further efforts to automate the process of harvesting mature embryos and process individual harvested embryos during desiccation for germination has been described in a series of patents (most recently in Swanda and Givens, 2017).

### Germination and Artificial Seeds

An idea that attracted much attention and praise from the traditional forestry industry is based on the concept of embedding the somatic embryos into an artificial seed. The goal is to allow storage and handling of the somatic embryo in the same way as the regular seed, which is anticipated to reduce the cost of adapting the handling technologies to somatic embryos. Artificial seed technology was extensively researched and different aspects supporting the concept developed (Sharma et al., 2013; Guan et al., 2016). Automated preparation of manufactured, artificial seeds followed by insertion of selected embryos is described in Hirahara (2009). A major unresolved problem with artificial seeds of woody species has been low and variable conversion rates to viable plants (Guan et al., 2016). Related to this are contamination problems caused by sucrose in the germination media.

An attractive alternative method to the artificial seed concept is however direct sowing of mature somatic embryos into a non-sterile environment. Such a method is claimed to be successful by placing embryos into an environment with specific humidity, temperature, nutrients, ambient light intensity and diurnal photoperiod, and shown to enable and facilitate germination (Polonenko et al., 2005). To date this has not been made to work on a practical scale.

### Germination and Planting

The steps following harvest of mature somatic embryos of conifers typically but not necessarily involve partial drying or desiccation of the mature embryos, followed by germination of the mature somatic embryo. Once the embryo is deemed to have sufficiently germinated by developing a root and a shoot, the germinant should be planted into a suitable compost. This process is considered another key rate-limiting step as the germinated embryos are fragile and often display different morphologies. Efforts to automate the process include an initial step of transferring the harvested mature embryos from the surface on which they were collected to germination medium by an automated process, and providing the necessary conditions for germination after transfer to the second surface (Jamruszka-Lewis and Starr, 2017).

In another recent effort to automate germination and planting, a system was developed that combines these steps through a connected process taking mature embryos harvested with the SE fluidics system through a germination platform allowing the mature embryos to be desiccated, germinated by contact with liquid medium, and then planted directly from the same position it was deposited after harvesting by the SE Fluidics system, as described in detail in Aidun and Egertsdotter (2018). The direct planting is performed by an automated planting system that is specifically designed to handle the germinated embryo from the germination platform after harvest with the SE Fluidics system (Aidun, 2016).

For carrot plant production from somatic embryos, a simplistic system utilizing a mist bioreactor was shown to work well simply by controlling the nutrient supply and aeration at each stage after a single set up of the apparatus, thus largely automating the whole SE process (Fei and Weathers, 2014).

Automated planting of seed into pots, and transplanting of plants from small pots to larger pots, are technologies that have been in use for a long time. Such automation can be applied to SE plants once the SE plant is established and growing *ex vitro*. However, for the steps before the SE plant is growing *ex vitro*, including the planting of the germinated mature embryo into compost, there is no known, well established automated technology currently in use for commercial SE plant production, although the Germination Platform technology developed as an add-on technology for the SE Fluidics system has the potential to handle this step.

Production of cuttings from SE-plants has been adopted on a larger scale for conifer species that are amenable to taking cuttings, notably *Picea sitchensis* (Thompson, 2013), *Pinus radiata* (Montalbán et al., 2011) and *Pinus taeda* (Schapovaloff and Raute, 2016). As there are no widely adopted automation procedures for production of cuttings, the use of two propagation techniques will further raise the costs due to additional manual labor.

In Sweden, millions of tree seedlings are produced every year of which 98% are of conifer species. Different companies deliver millions of seedlings from their nurseries to other forest companies and forest owners and this demand has been increasing over the last 15 years. Such huge numbers of seedlings make it imperative to automate the whole process. In Swedish forest nurseries the automated systems are in use for sorting seedlings with cameras, stacking, packaging, making pellet, transplanting, efficient seedling shipping and distribution. Different companies have adopted different methods of storing, packaging, and transplanting seedlings. Some companies have advantages over others in terms of efficient mechanizations, easy handling, cost effectiveness, and shorter delivery time (Ersson, 2016). The existence of such automated systems in the nursery sites will be helpful for implementation of SE plant production that is based on containerized plant production.

## State of the Art and Future Perspectives

As of today, no automated systems for large-scale production of somatic embryo plants are yet in commercial use. The SE Fluidics system supports singularization, selection and deposition of mature somatic embryos and can enable large-scale production of SE plants of conifers by overcoming the labor-intensive steps of harvesting and separating good mature embryos from the culture mix of mature embryos and non-responsive PEMs. Such a system is planned to become the core of a full commercial system for SE plant production of Norway spruce in Sweden. Further development of the GP system and vertical germination as described in Aidun and Egertsdotter (2018) holds promise for generating better-quality germinants coupled with the option to automated planting into the non-sterile substrate. It however appears that the immediate future for applying SE for large-scale plant production is based on selection of cell lines permissive to the SE process, from a very large number of improved seeds. Numerous attempts to optimize the medium and growth conditions have not made significant differences, but finding the “best” cell lines for the existing methods in the labs greatly enhances the results. Furthermore, improving the selection criteria for “good” mature embryos will improve the downstream yields of plants. Efforts have been made to design a classification-system for plant embryos that uses a logistic regression model based on image or spectral data correlated with various quality aspects (Timmis and Toland, 2009). The possibilities to utilize spectral imaging have recently been demonstrated for analyses of chemical composition of intact maize kernels (Yang et al., 2018). Furthermore, the future perspective of utilizing hyperspectral imaging has the possibility to further distinguish detailed characteristics of mature embryos likely to germinate and form plants (Lu and Fei, 2014). In *Theobroma cacao*, transformants with genes controlling transcription factors show greatly enhanced SE performance (Shires et al., 2017) and references therein.

## Author Contributions

UE edited and wrote the bulk of the manuscript, and directed co-authors. IA and DC contributed to parts of the manuscript writing. DC edited and checked the language.

### Conflict of Interest Statement

The authors declare that the research was conducted in the absence of any commercial or financial relationships that could be construed as a potential conflict of interest.
